# Euglycemic Diabetic Ketoacidosis in Concurrent Very Low-carbohydrate Diet and Sodium-glucose Transporter-2 Inhibitor Use: A Case Report

**DOI:** 10.5811/cpcem.2020.2.45904

**Published:** 2020-04-23

**Authors:** Matthew Earle, Brian Ault, Caitlin Bonney

**Affiliations:** University of Nevada Las Vegas, Department of Emergency Medicine, Las Vegas, Nevada

**Keywords:** SGLT-2 Inhibitors, Ketoacidosis, Low-carbohydrate, Ketogenic

## Abstract

**Introduction:**

With the incredibly high incidence of Type 2 Diabetes in the current population of emergency department patients, it is critical for clinicians to understand the possible complications of the treatment of this disease. Medication like canagliflozin are more common to encounter on patient’s home medication lists and clinicians should be aware of how these medications, alone or combined with dietary modifications, can result in significant pathology and even mortality if not appropriately treated.

**Case Report:**

We report a case of a patient with type II diabetes mellitus who presented with euglycemic diabetic ketoacidosis in the setting of concurrent use of canagliflozin, a sodium-glucose transporter-2 (SGLT-2) inhibitor, and strict adherence to a low-carbohydrate ketogenic diet for weight control.

**Discussion:**

Euglycemic ketoacidosis has previously been observed in both diabetic and non-diabetic patients following strict ketogenic diets, as well as in diabetic patients being treated with SGLT-2 inhibitors.

**Conclusion:**

As more patients choose ketogenic diets for weight control and diabetes management, clinicians should be aware of this potentially life-threatening complication in patients concurrently taking SGLT-2 inhibitors.

## INTRODUCTION

Very low-carbohydrate, or ketogenic, diets originated in the realm of fad weight-loss diets, but the practice has moved into the armamentarium of medicine in controlling weight, epilepsy, and diabetes. Sodium-glucose transporter-2 (SGLT-2) inhibitors have also become more heavily used in the control of diabetes, leading to an increased incidence of concomitant use of ketogenic diets and SGLT-2 inhibitors to control weight in diabetic patients.^1^,^2^ Given that both ketogenic diet and SGLT-2 inhibitors alone can result in euglycemic ketosis,^3^,^4^ there is likely an increased risk of developing this derangement when the diet and medication are combined. We report here the case of a patient who developed life-threatening euglycemic ketosis while adhering to a strict ketogenic diet with concomitant canagliflozin use.

## CASE REPORT

A 31-year-old female with a history of type II diabetes mellitus (T2DM) presented to a primary care office with dizziness and shortness of breath worsening over one week. She also noted slurring of her speech, nausea, pain radiating down the posterior aspect of both legs, and constipation. She had no history of previous episodes of diabetic ketoacidosis (DKA) or hyperosmolar hyperglycemic state (HHS). The patient had been attempting to control her T2DM by following a very low-carbohydrate, ketogenic diet for about two weeks, during which she had restricted her carbohydrate intake to 10–15 grams per day. Fluid intake included 3–4 liters of water per day and pickle juice. Over this time, she had a 12.2 kilogram weight loss. She had previously used long-acting insulin (insulin detemir) to control her glucose but had not needed to use insulin to maintain normoglycemia in weeks and was not using insulin at presentation. She stopped using insulin at the time of starting the ketogenic diet.

At the time of presentation, her T2DM management regime consisted solely of canagliflozin and dietary modifications. The patient initially presented to her primary care physician, where she was found to have a blood glucose of 133 milligrams per deciliter (mg/dL) on fingerstick and positive urine ketones. She was sent to the emergency department (ED) for further evaluation. Initial vital signs in the ED were blood pressure 126/78 millimeters of mercury, pulse 137 beats per minute, temperature 97.7º Fahrenheit (36.5º Celsius), respirations 24 breaths per minute, and oxygen saturation 100% on room air. On physical exam, the patient appeared acutely ill. Neurological exam on presentation was notable for Glasgow Coma Scale of 15, drunken affect, gait instability, and mildly slurred speech without aphasia.

Initial electrocardiogram showed sinus tachycardia with normal intervals. Chest radiograph was normal. Laboratory evaluation showed a pH on a venous blood gas of 7.056 with a bicarbonate of 8.0 milliequivalents per liter (mEq/L), blood glucose of 139 mg/dL, blood ketones of 80 mg/dL, lactate of 1.4 millimole per liter (mmol/L), and an anion gap of 29. The remainder of lab results are displayed in the Table. The patient was treated with a bolus of one L of lactated Ringer’s solution, followed by an additional of one L 5% dextrose (D5) normal saline and an intravenous potassium bolus of 20 mEq. After administration of approximately 200 milliliters of D5-containing solution, the patient had normalization of her neurological deficits, with no further speech slurring or feelings of intoxication. An insulin infusion was not started emergently, in order to facilitate transfer to an appropriate facility that could provide intensive care unit (ICU) admission and management.

The patient was transferred for admission to ICU level of care for severe metabolic acidosis, ketosis, and tachycardia. The patient had an uneventful course over the next few days with normalization of her ketones and acidosis while being treated with an insulin infusion with concomitant glucose-containing fluids to maintain euglycemia. She was discharged at baseline health and was lost to follow-up.

## DISCUSSION

This case demonstrates the occurrence of euglycemic ketoacidosis in a patient with T2DM concurrently following a low-carbohydrate, ketogenic diet and using an SGLT-2 inhibitor. This is an uncommon cause of altered mental status, and it is important for emergency physicians to be aware of this potential complication of diabetes management.

**CPC-EM Capsule**

What do we already know about this clinical entity?

*Euglycemic diabetic ketoacidosis (EDKA) and its relation to* sodium-glucose transporter-2 (*SLGT-2) inhibitors has been previously acknowledged and cases reported.*

What makes this presentation of disease reportable?

Our case represents a novel combination of extreme EDKA in a patient on an SLGT-2 inhibitor in combination with a very-low carbohydrate (or ketogenic) diet.

What is the major learning point?

Ketogenic diets and SGLT-2 inhibitor use, both singly and in combination, can lead to severe, life-threatening EDKA.

How might this improve emergency medicine practice?

Clinicians should consider EDKA in acidotic patients for whom new dietary trends can lead to significant medication interactions and morbidity.

Canagliflozin is an oral sodium-glucose cotransporter-2 (SGLT-2) inhibitor approved by the United States Food and Drug Administration (FDA) for treatment of T2DM and has been shown to improve glycemic control, weight loss, and hemoglobin A1c levels. SGLT-2 inhibitors decrease serum glucose concentrations by preventing glucose reabsorption in the proximal renal tubule of the kidney, thereby promoting glucosuria. SGLT-2 inhibitors also stimulate glucagon secretion from the alpha cells of the pancreas, minimizing the potential for hypoglycemic events. Although SGLT-2 inhibitors have shown efficacy in diabetes management, serious adverse events have been associated with their use. In 2017 the FDA initiated a boxed warning regarding the increased risk of leg and foot amputations in patients taking canagliflozin. Large surveillance studies have also noted increased risk of diabetic ketoacidosis with SGLT-2 inhibitors.^4^

Euglycemic diabetic ketoacidosis (EDKA) is a previously rare clinical condition now showing increasing prevalence with the use of SGLT-2 inhibitors. The pathogenesis of EDKA involves an increased glucagon to insulin ratio, in which ketogenesis is stimulated without hyperglycemia. Increased glucagon levels contribute to gluconeogenesis, glycogenolysis and fatty acid metabolism to mobilize energy substrates.^5^ In diabetic patients, however, the relative shortage of insulin precludes intracellular glucose utilization, further exacerbating metabolic acidosis. SGLT-2 inhibitors promote ketoacidosis by increasing glucagon secretion and limit available circulating glucose by promoting glucosuria. SGLT-2 inhibitors also exacerbate the osmotic diuresis of ketoacidosis. Normally, glucosuria occurs when blood glucose concentrations exceed 225 mg/dL. SGLT-2 inhibitors block reabsorption of glucose in the proximal renal tubule even at physiologic serum glucose concentrations and cause glucosuria and osmotic diuresis even in normoglycemia.

Low-carbohydrate, ketogenic diets significantly restrict the dietary supply of carbohydrates, promoting a ketogenic state of fatty acid metabolism. Although ketogenic diets induce a state of ketosis, they are not commonly associated with diabetic ketoacidosis.^6^,^7^ However, a previous case of ketoacidosis during a low-carbohydrate diet has been reported.^8^ Treatment for EDKA is not fundamentally different from the treatment of hyperglycemia DKA, using fluid resuscitation and insulin administration as the mainstays of therapy.^9^,^10^ However, patients will likely require earlier administration of glucose-containing fluids and should have the SGLT-2 inhibiting medication held. At discharge, consideration should be given to discontinuing the SGLT-2 inhibitor.

Since both low-carbohydrate, ketogenic diets and SGLT-2 inhibitors increase glucagon secretion while limiting serum glucose levels, a synergistic effect increasing the risk for EDKA is plausible. Cases of severe ketoacidosis associated with a low-carbohydrate diet and use of ipragliflozin and dapagliflozin have previously been reported.^11^,^12^ Low-carbohydrate/high-fat meals stimulate glucagon production and fatty acid metabolism while limiting serum glucose availability. This physiologic state is likely exacerbated by the concurrent glucagon upregulation and depletion of serum glucose caused by SGLT-2 inhibitors, leading to severe acidosis with ketosis. The limited supply of dietary carbohydrates combined with SGLT-2 inhibitor-induced glucosuria may create a state of diabetic ketoacidosis without elevated serum glucose. This association will require further study for validation.

The patient’s initial altered mental status and speech changes may be attributable to her relative depletion of intracellular and intravascular glucose stores. Similar changes in mental status in patients with alcoholic ketoacidosis (AKA) have been previously attributed to intracellular hypoglycemia. As discussed above, this patient also experienced relative hypoglycemia due to her low relative insulin levels, creating a similar state as that of AKA.^13^ Thus, the treatment with the glucose-containing fluids likely terminated the patient’s neurologic symptoms by resolving her relative hypoglycemia.

## CONCLUSION

Euglycemic diabetic ketoacidosis is a recognized complication of SGLT-2 inhibitor use for weight and glucose control in patients with diabetes. However, there have been few if any previous published cases that were complicated by a concomitant ketogenic diet. Our goal is not to defame the practice of using ketogenic diets in an effort to control body weight and blood glucose, but to caution clinicians that ketogenic diets and SGLT-2 inhibitor use, both singly and in combination, can lead to severe, life-threatening EDKA.

## Figures and Tables

**Table:** Initial laboratory values of patient who presented to the emergency department with dizziness, shortness of breath, and slurred speech.

Lab	Value	RR	Units
Sodium	139	136–145	mEQ/L
Potassium	3.5	3.5–5.1	mEQ/L
Chloride	102	98–107	mEQ/L
Carbon Dioxide	8	22–30	mEQ/L
Anion Gap	29	3–11	NA
BUN	12	7–18	mg/dL
Creatinine	0.85	0.6–1.3	mg/dL
Glucose	139	70–99	mg/dL
Lactate	1.4	0.4–2.0	mMol/L
Serum Osmolality	295	280–295	mOSM/kg
Ammonia	27	<32	μMol/L
β-Hcg	NEG	NA	NA
Total Protein	8	6.4–8.2	g/dL
WBC	10.6	4–10	k/mm3
Hemoglobin	16.1	11–15	g/dL
Platelets	287	150–400	k/mm3
INR	0.8	NA	NA
Urine Ketones	80	NEG	mg/dL
Urine Glucose	500	NEG	mg/dL
Urine Blood	NEG	NEG	NA
Urine Protein	100	NEG	mg/dL

*RR*, reference range; *mEQ*, milliequivalents; *NA*, not applicable; *BUN*, blood urea nitrogen; *mg*, milligrams; *dL*, deciliter; *mMol*, millimoles; *L*, liter; *mOSM/kg*, milliosmoles per kilogram; *μMol*, micromoles; *β-Hcg*, human chorionic gonadatropin beta-subunit; *NEG*, negative; *g*, gram; *WBC*, white blood count; *K/mm^3^*, thousand per cubic millimeter; *INR*, international normalized range.
